# Magnetic ZnO Crystal Nanoparticle Growth on Reduced Graphene Oxide for Enhanced Photocatalytic Performance under Visible Light Irradiation

**DOI:** 10.3390/molecules26082269

**Published:** 2021-04-14

**Authors:** Rania Elshypany, Hanaa Selim, K. Zakaria, Ahmed H. Moustafa, Sadeek A. Sadeek, S.I. Sharaa, Patrice Raynaud, Amr A. Nada

**Affiliations:** 1 Department of Analysis and Evaluation, Egyptian Petroleum Research Institute, Nasr City, Cairo 11727, Egypt; raniasalem372@yahoo.com (R.E.); hanaaselimali@yahoo.com (H.S.); khaled209eg@yahoo.com (K.Z.); sishara.epri@yahoo.com (S.I.S.); 2 Department of Chemistry, Faculty of Science, Zagazig University, Zagazig 44519, Egypt; ah_hu_mostafa@yahoo.com (A.H.M.); sadeekasadeekzag@yahoo.com (S.A.S.); 3 Laboratoire Plasma et Conversion d’Energie (LAPLACE), Université de Toulouse, CNRS, INPT, UPS, 31062 Toulouse, France; raynaud@laplace.univ-tlse.fr

**Keywords:** crystal magnetite, ZnO, reduced graphene oxide, photocatalytic activity

## Abstract

Magnetite zinc oxide (MZ) (Fe_3_O_4_/ZnO) with different ratios of reduced graphene oxide (rGO) was synthesized using the solid-state method. The structural and optical properties of the nanocomposites were analyzed using transmission electron microscopy (TEM), X-ray diffraction (XRD), Raman spectroscopy, Fourier-transform infrared spectroscopy (FTIR), ultraviolet–visible diffuse reflectance spectroscopy (UV–Vis/DRS), and photoluminescence (PL) spectrophotometry. In particular, the analyses show higher photocatalytic movement for crystalline nanocomposite (MZG) than MZ and ZnO nanoparticles. The photocatalytic degradation of methylene blue (MB) with crystalline ZnO for 1.5 h under visible light was 12%. By contrast, the photocatalytic activity for MZG was more than 98.5%. The superior photocatalytic activity of the crystalline nanocomposite was detected to be due to the synergistic effect between magnetite and zinc oxide in the presence of reduced graphene oxide. Moreover, the fabricated nanocomposite had high electron–hole stability. The crystalline nanocomposite was stable when the material was used several times.

## 1. Introduction

Wastewater resulting from various industries such as the leather, paper, and textile industries can contain various dyes [1]. The production of dyes is a major concern because most of them are extremely dangerous and hazardous to the environment as well as human health [2,3]. To date, many traditional water treatment technologies have been implemented, including adsorption, chemical precipitation, and chemical oxidation; nonetheless, their success has proved to be limited [2]. However, these types of treatment methods have resulted in the production of secondary pollutants and significant associated health problems [4].

Due to the problems of conventional water treatment methods, the photocatalytic oxidation process has become a fundamental area in the field of the wastewater treatment technologies [2,5], as it leads to a complete degradation of organic pollutants into carbon dioxide and water [6]. From a chemical perspective, the photogenerated charge carriers in crystalline semiconductors are allowed to produce highly reactive chemical species, such as hydroxyl radicals, which may mineralize a wide range of organic contaminants rapidly and nonselectively [7,8].

Zinc oxide (ZnO) is one of the most well-known crystalline photocatalysts used in wastewater purification processes, as it is not only nontoxic but is also readily available, has suitable energy band positions, and has high excitonic stability [9,10,11,12]. Despite its safety, stability, and availability, ZnO has multiple limitations, such as a band gap of perfect ZnO that is too large (3.37 eV) and cannot exhibit visible-light photocatalytic activity, low efficiency due to the decreased ZnO photocatalytic activity, and fast recombination of the hole–electron pairs and photocorrosion arising from the dissolution of ZnO into Zn^2+^ ions in aqueous solutions under UV irradiation [13,14,15,16,17,18,19,20]. Several studies have attempted to prevent these limitations in order to modify ZnO properties using methods such as (a) defect engineering, which is often employed to regulate the band structures shifting to visible light adsorption, and (b) modification via combination with carbon materials, nonmetal doping, transition, and noble metal doping, as well as support ZnO with reduced graphene oxide (rGO) [21,22,23,24,25,26]. Those methods enhance the photocatalytic performance of ZnO through minimizing the band gap absorbing in the visible region to generate excellent visible-light photocatalytic activities, restraining e^−^/h^+^ recombination and suppressing the photocorrosion of ZnO.

Reduced graphene oxide plays a vital role as a good support with which to achieve a uniform distribution of ZnO without aggregation. Despite the high photocatalytic activity of rGO-ZnO nanocomposites, there is an increasing impediment due to the difficulty of recycling them. Many of the previous and current research studies investigating the issue of recycling nanocomposite photocatalysts have been more concerned with the incorporation of these photocatalysts with magnetic nanoparticles, Fe_3_O_4_ [27,28,29,30,31]. The criteria for selecting the magnetic nanoparticles include high surface area due to the increased durability and ability to prevent the agglomeration of the catalysts, in addition to being easily isolated with an external magnetic field from water [32].

In the present work, crystalline magnetite ZnO was synthesized with different ratios of graphene oxide using a facile and fast method: a homogeneous sonication method. This method was used at an ambient temperature without any further modification. This method was used before that for the fabrication of the trio nanocomposite Fe_3_O_4_/rGO/ZnO. This nanocomposite exhibited high efficiency with high stability for the degradation of organic pollutants. The crystal structure and optical properties of the resulting photocatalyst Fe_3_O_4_/rGO/ZnO were confirmed using TEM, XRD, Raman spectroscopy, FTIR, XPS, VSM, UV–Vis/DRS, and PL spectrophotometry. The photocatalytic performance of Fe_3_O_4_/rGO/ZnO was examined by its ability to degrade methylene blue (MB) under visible light irradiation.

## 2. Results and Discussion

### 2.1. Characterization Analysis

**Figure 1:**
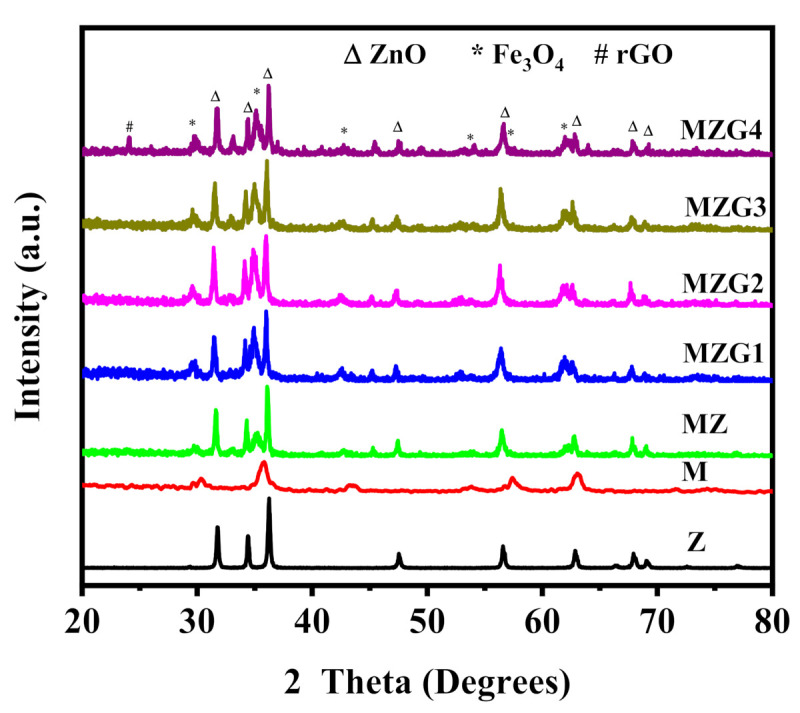
XRD spectra of Z, M, MZ, MZG1, MZG2, MZG3, and MZG4 nanoparticles (NPs).

The crystallinity and the diffraction patterns of magnetic zinc oxide nanoparticles that were loaded on reduced graphene oxide are illustrated in Figure 1. The pattern of Z sample (ZnO) has nine characteristic peaks at 2Ɵ = 31.74°, 34.26°, 36.21°, 47.56°, 56.49°, 62.67°, 66.06°, 67.8°, and 69.03°, which were indexed to the (100), (002), (101), (102), (110), (103), (200), (112), and (201) Miller indices, respectively. The diffractions peaks were recorded in the Z sample according to ZnO crystals with a hexagonal wurtzite structure (JCPDS Card No. 01-080-4199) [33]. The strong sharp peaks suggest that the prepared ZnO NPs have high crystallinity [34]. By contrast, the M sample (Fe_3_O_4_) had six characteristic peaks (2Ɵ = 29.91°, 35.36°, 43.02°, 53.33°, 56.98°, and 62.48°) according to the Miller indices (220), (311), (400), (422), (511), and (440), respectively. All of the observed peaks in the M sample were attributed to the cubic spinal structured magnetite (JCPDS Card no. 01-089-1397) [35]. In the curve of Fe_3_O_4_/ZnO (MZ), the peaks of both magnetite and zinc oxide were detected. In the curves of MZG1, MZG2, MZG3, and MZG4 (Fe_3_O_4_/ZnO with different weight ratios of GO of 0.5, 1, 1.5, and 2 wt %, respectively), all the diffraction peaks corresponding to Fe_3_O_4_ and ZnO crystals were exposed. In the status of higher graphene content (MZG4), a small peak belonging to reduced graphene oxide was detected at 2 theta ~24.11°, which was identical to the d spacing of ~0.369 nm [36]. The Debye-Scherrer equation was used to calculate the crystallite sizes of the prepared nanocomposites (Equation (1)). The average crystallite sizes of MZ, MZG1, MZG2, MZG3, and MZG4 were 33.5, 33.1, 32.9, 33, and 32.8 nm, respectively. The crystallite sizes of the prepared nanocomposites were detected in the range 33.5 to 32.8 nm. This indicated the crystallite size of Fe_3_O_4_/ZnO wasn’t changed after loading with rGO [36].

d = kλ/βcosθ(1)

where d is the mean particle diameter, assuming spherical particles, k is the Scherer constant (= 0.9), λ is the wavelength of the X-ray beam (1.5405 Å), β is the full width at half maximum (FWHM) of the diffracted peak, and θ is the angle of diffraction.

**Figure 2:**
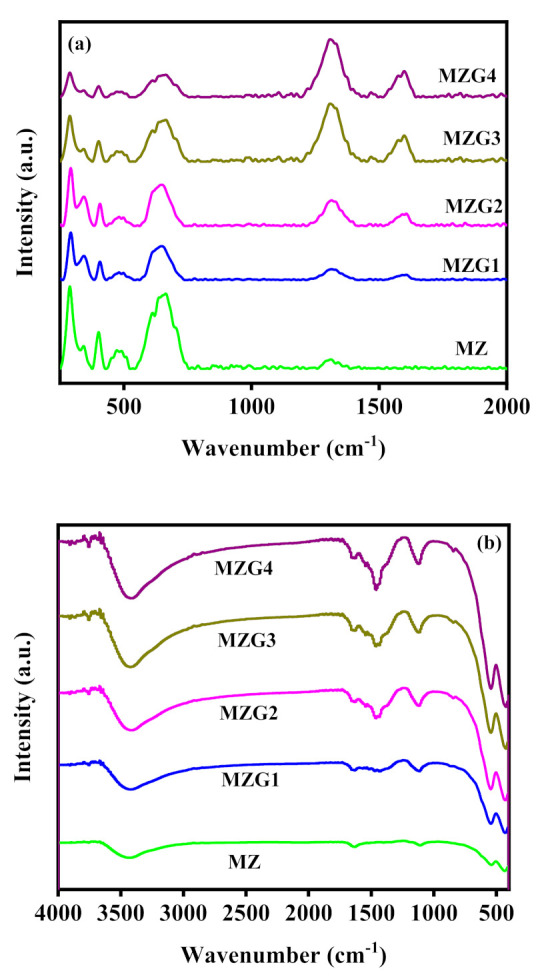
Raman (**a**) and FTIR (**b**) spectra of MZ, MZG1, MZG2, MZG3, and MZG4 crystalline nanocomposites.

Raman spectroscopy is an important technique for the description of graphitic-based materials. The Raman spectrum of ZnO in MZ samples shows peaks at 472 cm^−1^ (E_2H_ mode) as presented in Figure 2a. The formation of the Fe_3_O_4_/ZnO composites causes a kind of charge transfer. It causes a vibrational frequency shift leading to a Raman blueshift approximately 37 cm^−1^ beyond pure ZnO as illustrated in our previous studies [37,38]. By contrast, Fe_3_O_4_ has redshift in bands at 343 and 663 cm^−1^ [39]. Moreover, there were other bands observed at 290 and 400 cm^−1^ corresponding to Fe_3_O_4_ [40]. In addition, the band was observed at 1312 cm^−1^ in the MZ samples, which was corresponding to the two magnons scattering. This scattering was produced from the antiparallel spin of two magnons [39]. In the presence of graphene, the distinctive bands of graphene oxide (D and G bands at 1340 and 1577 cm^−1^, respectively) appeared as presented in Figure 2a and Figure S1. The D and G bands provide valuable information of the defect in sp^2^ carbon atoms [41]. The intensity ratio between the D and G bands is significant for indicating the reduction in graphene oxide. In the GO sample, the I_D_/I_G_ was 0.88. However, the I_D_/I_G_ in the cases of MZG1, MZG2, MZG3, and MZG4 was 1.35, 1.37, 1.43, and 1.44, respectively. This indicated that the structure of GO changed in the presence of Fe_3_O_4_/ZnO to reduced graphene oxide [42]. The I_D_/I_G_ ratios in MZG3 and MZG4 were near to each other due to the reduction of graphene oxide related to the ratio between Fe_3_O_4_/ZnO and GO [36], whereas according to the Raman analysis, the best ratio between Fe_3_O_4_/ZnO and GO for achieving a high reduction of graphene oxide was in MZG3. However, MZG4 had enrichment of graphene layers, which led to a stabilization of or a decrease in the reduction process and an accumulation of graphene layers in nanocomposite. Thus, the Raman results were in accordance with the XRD results.

The FTIR spectra of the crystalline nanocomposites (MZ, MZG1, MZG2, MZG3, and MZG4) are shown in Figure 2b. The bands at 440 and 544 cm^−1^ were assigned to the formation of ZnO and Fe_3_O_4_ as Zn-O and Fe-O bonds, respectively [31], while the broad bands at 3440 and 1640 cm^−1^ were assigned to the stretching and bending modes of the hydroxyl group of H_2_O. In addition, the band at 1460 cm^−1^ corresponded to M-O-G vibration [43], where M is Fe or Zn, which confirmed the interaction between Zn, Fe, and C, and thus the loading of Fe_3_O_4_, ZnO, and rGO [43].

**Figure 3:**
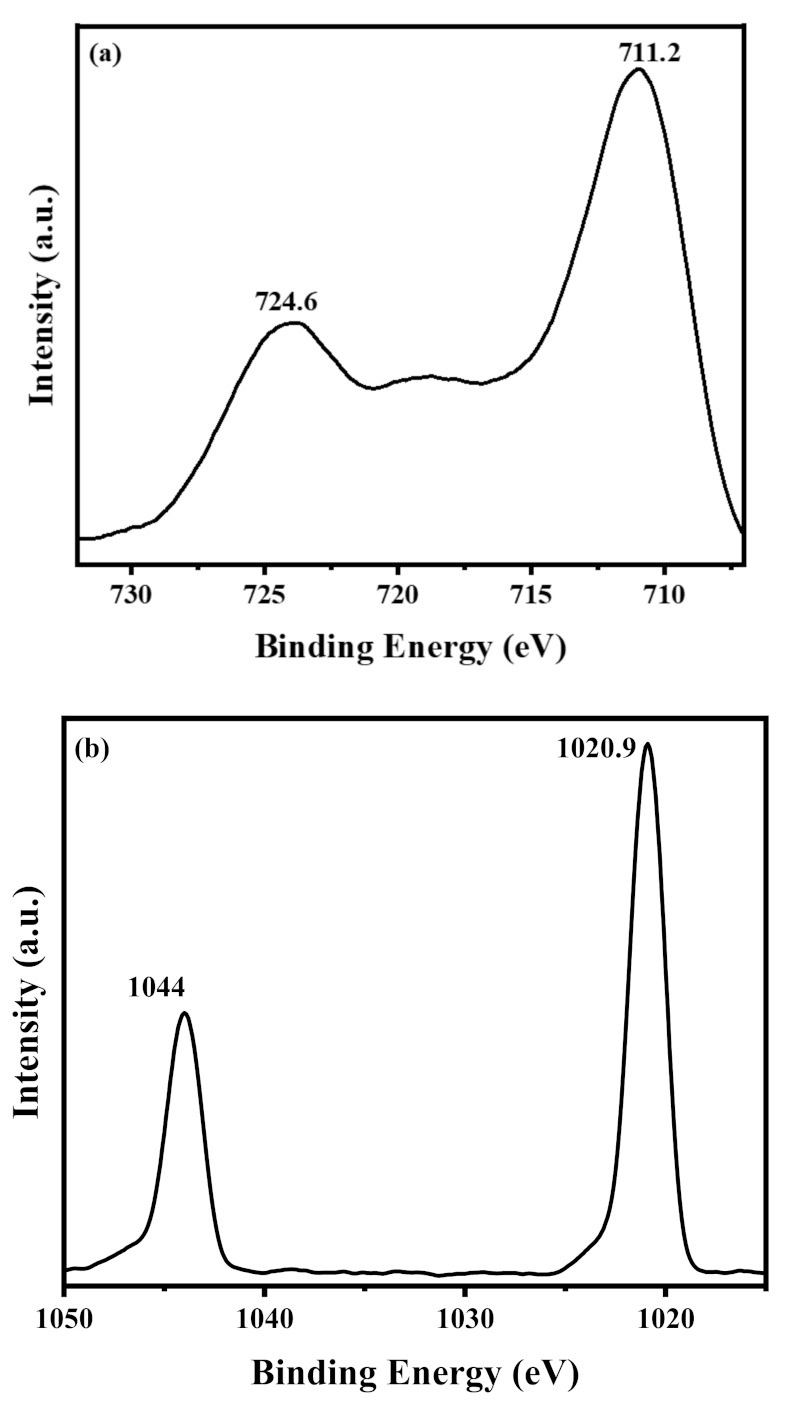

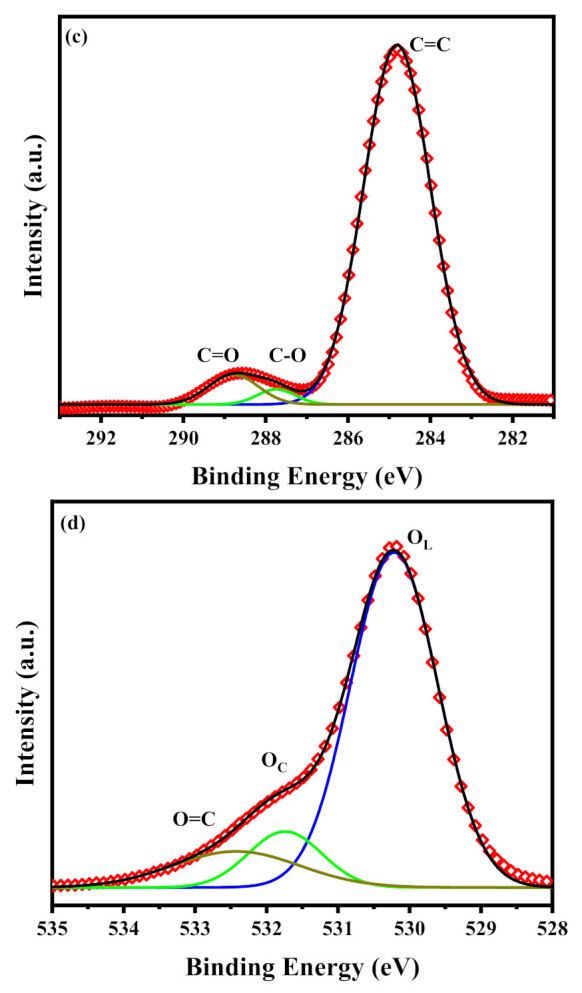
(**a**) Fe 2p, (**b**) Zn 2p, (**c**) C 1s, and (**d**) O 1s XPS spectra of MZG3 crystalline nanocomposites.

Further information on the chemical composition of the MZG3 nanocomposite was detected using X-ray photoelectron spectroscopy analysis (XPS), as shown in Figure 3a,b. The XPS spectra of Fe 2p in Figure 3a can be presented with two peaks at ∼711.2 and 724.6 eV corresponding to 2p_3/2_ and 2p_1/2_, respectively, without satellite peaks [44]. These peaks referred to Fe_3_O_4_ matched with the XRD. As shown in Figure 3b, the binding energy at 1020.9 eV and 1044 eV was related to Zn 2p_3/2_ and 2p_1/2_ [45]. The C 1s XPS spectra are presented in Figure 3c. They can be fitted with three peaks at ~284.8, 287.7, and 288.8 eV for (C=C group), (C-O group), and (C=O group), respectively [46]. The ratio between the C-C and (C-O and C=O) peaks could indicate the reduction process. This ratio was found to be 11.7 for MZG3, which is high enough to clarify the complete reduction process for rGO in the nanocomposite [46]. The O 1s XPS spectra in Figure 3d can be fitted as three peaks. The peaks at ~530.2, 531.7, and 532.4 eV correspond to lattice oxygen (O_L_), chemisorbed oxygen (O_C_), and an O=C bond in the Fe_3_O_4_/rGO/ZnO nanocomposite [47].

The VSM technique was used to detect the magnetic behavior of Fe_3_O_4_ and MZG3 nanocomposite and presented in Figure S2. The magnetization from hysteresis loops for the M and MZG3 nanocomposites were 59.6 and 35.4 emu/g, respectively. The recorded magnetization values indicate the ability to use external magnets for photocatalyst separation from the solution and to utilize the nanocomposite several times.

**Figure 4:**
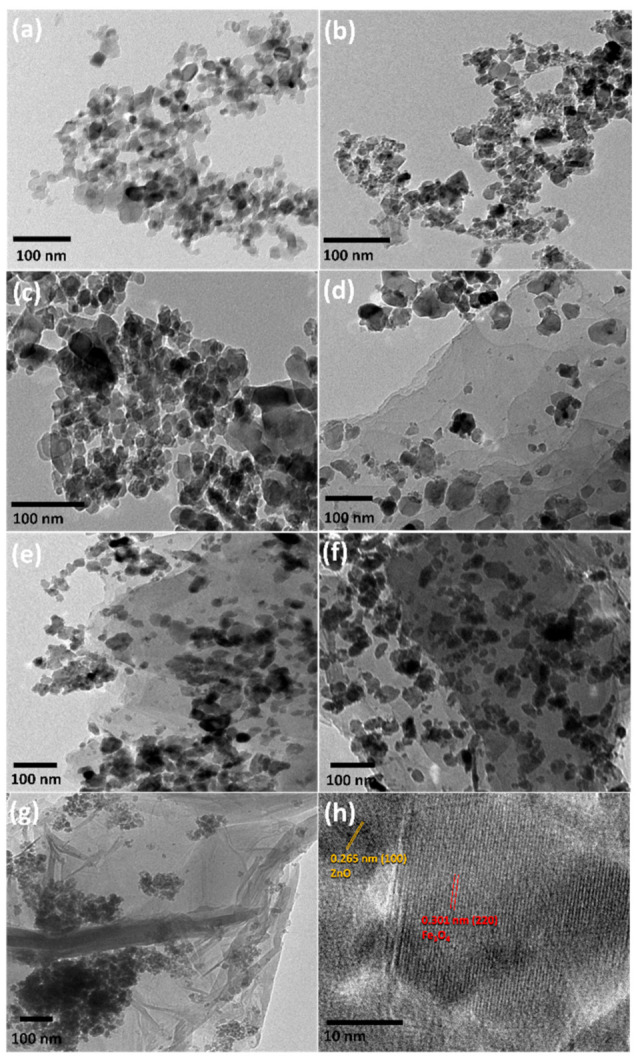
TEM images of Z (**a**), M (**b**), MZ (**c**), MZG1 (**d**), MZG2 (**e**), MZG3 (**f**), MZG4 (**g**), and a high-resolution transmission electron microscope (HRTEM) image of MZG3 (**h**).

The morphology of the elaborated nanocomposites was detected using TEM as presented in Figure 4. It was found that the distribution of Fe_3_O_4_/ZnO (MZ) nanocomposites on the sheets of reduced graphene oxide depends on the ratio between rGO and MZ. The TEM images of the MZGx nanocomposites indicated the presence of a two-dimensional structure of rGO with nanoparticles of Fe_3_O_4_/ZnO, which were well dispersed on the graphene sheet [48,49]. Moreover, we can see that MZG3 (Figure 4f) presented a homogeneous distribution. By contrast, MZG4 (Figure 4g) had more aggregation of rGO. All of this information is consistent with the XRD and Raman sections. Moreover, the MZG3 nanocomposite was detected using high-resolution transmission electron microscopy (HRTEM) as presented in Figure 4h. The d-lattice spacing was detected as 0.265 ± 0.05 nm and 0.301 ± 0.04 nm for (100) ZnO and (220) Fe_3_O_4_, respectively [50,51]. Consequently, the HRTEM result shows that the ZnO and Fe_3_O_4_ were tightly connected to each other.

**Figure 5:**
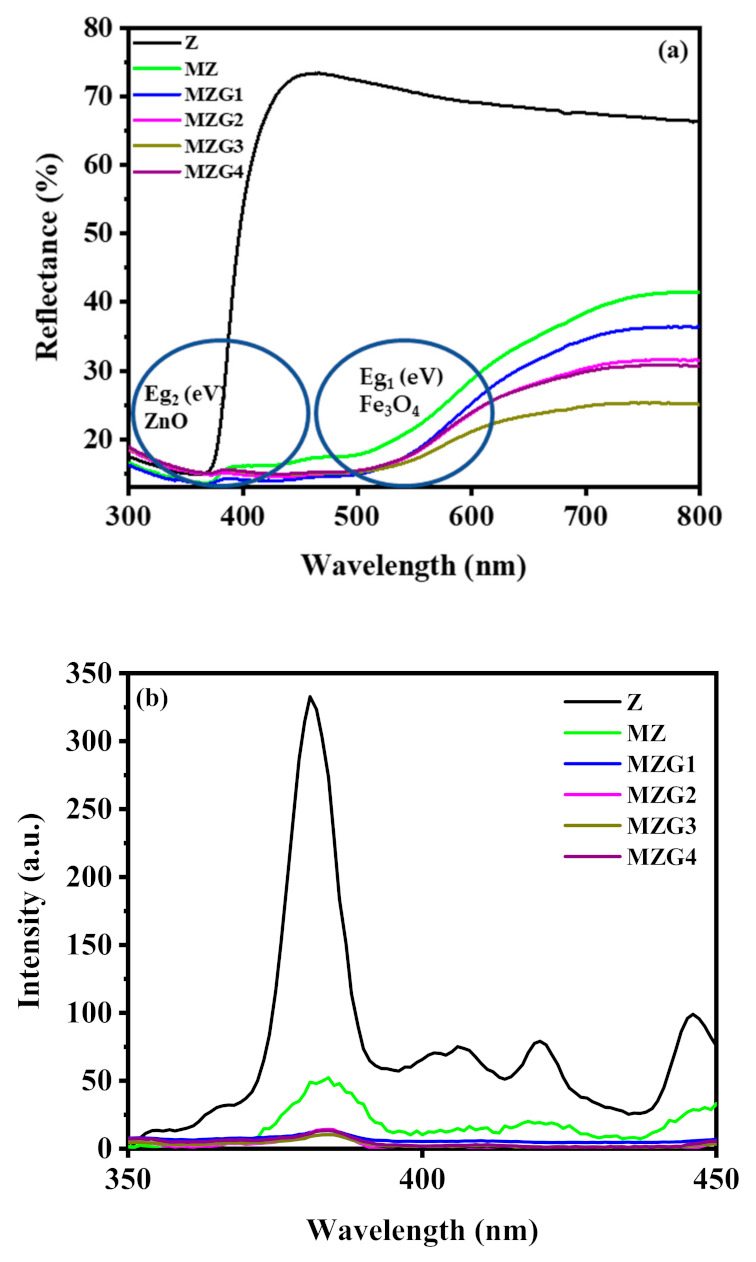
(**a**) Diffuse reflectance spectra and (**b**) photoluminescence spectra of Z, MZ, MZG1, MZG2, MZG3, and MZG4 crystalline nanocomposites.

The optical properties of the prepared crystalline nanocomposites were investigated using UV–Vis DRS (Figure 5a) and PL (Figure 5b) at room temperature. The diffuse reflectance spectra of the prepared samples were investigated using UV–Vis optical spectroscopy in the range of 200–800 nm. The optical band gaps were calculated from the following equation:

(2) $$\alpha hv=A{\left(hv-Eg\right)}^{n/2}$$

where v is the light frequency, *α* is the absorption coefficient, and *n* is the constant of proportionality. Notice that *n* = 1 for the direct transition in the prepared nanocomposite.

The crystalline nanocomposites had a redshift in the presence of rGO, especially for E_g1_ (corresponding to Fe_3_O_4_), as illustrated in Table 1. This shift was due to the bond of M (Fe or Zn) with graphene (M-O-C), this having been deduced from the result of FTIR [36]. MZG3 presented the lowest intensity of reflectance spectra. This is probably due to the high absorption of light in the case of MZG3 compared to that of other samples [36]. By contrast, MZG4 had the highest amount of reduced graphene layers, which led to the accumulation of graphene and prevented light passing to the nanocomposite as observed in the TEM image. Moreover, the presence of rGO with two band gaps led to more stability between the electron and hole pairs under visible light.

The photoluminescence spectra of Z, MZ, MZG1, MZG2, MZG3, and MZG4 at room temperature are presented in Figure 5b. The Z sample for bare ZnO has a strong emission band at 381 nm with some bands at 406, 420, and 445 nm due to deep level emission (DLE) [52]. Moreover, there was a synergistic effect due to the presence of Fe_3_O_4_ and rGO with ZnO as a triple nanocomposite to a drastic quenching of PL intensity, which increased the lifetime of electron stability.

### 2.2. Photocatalytic Activity of the MZG Crystalline Nanocomposite

**Figure 6:**
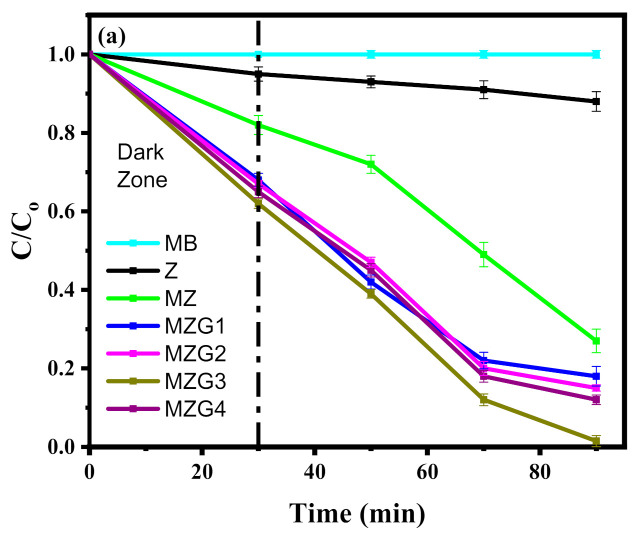

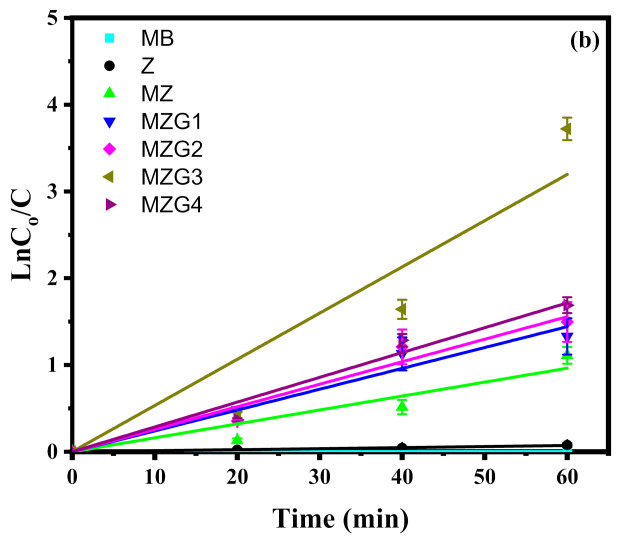
(**a**) Photocatalytic degradation and (**b**) kinetic photodegradation of methylene blue (MB) by no catalyst (MB), Z, MZ, MZG1, MZG2, MZG3, and MZG4 crystalline nanocomposites.

The photocatalytic degradation activity of Fe_3_O_4_/rGO/ZnO nanocomposites was investigated for the degradation of MB dye under visible light irradiation. In Figure 6a, the photodegradation efficiency for the organic pollutant (MB) of MZGx is presented. The highest degradation activity was recorded for MZG3. It was detected that the activity of the crystalline nanocomposites depended on the amount of reduced graphene oxide, up to a certain ratio (MZG3), which led to the higher efficiency in the transportation of the photogenerated charge carriers of rGO [53,54]. MZG4 had a higher amount of rGO; however, this led to a decrease in catalytic activity. This behavior is attributed to the excess of rGO layers covering the nanoparticles that hinder the propagation of the light. The aggregation of rGO layers was detected and confirmed by TEM and UV–DR.

The kinetics of MB photodegradation by the prepared nanocomposites were evaluated by a Langmuir–Hinshelwood (L–H) first-order kinetics model. The equation of a L–H kinetics model can be expressed as

*R* = d*C/*d*t* = *kKC/*(1 + *KC*) (3)

where *R* is the MB degradation rate (mg L^−1^ min^−1^), *C* is the MB concentration (mg L^−1^), *t* is the irradiation time, *k* is the reaction rate constant (mg L^−1^ min^−1^), and *K* is the MB adsorption coefficient (mg L^−1^). The photodegradation reaction of MB follows a pseudo-first-order kinetic [37,55]:

Ln (*C*_0_/*C*) = *kKT* = *k*_a_*t* (4)

where *k*_a_ is the constant rate (min^−1^), *C*_0_ is the initial concentration (mg L^−1^), and *C* is the MB concentration at time *t*. *k*_a_ was obtained from the linear relation between ln (*C*_0_/*C*) and time as presented in Figure 6b. Thus, the values of k_a_ for each sample are given in the following increasing order: MZG3 (0.0533 min^−1^) > MZG4 (0.0286 min^−1^) > MZG2 (0.0259 min^−1^) > MZG1 (0.0241 min^−1^) > MZ (0.0161 min^−1^) > Z (0.0012 min^−1^) > MB (2.1 × 10^−8^ min^−1^) with *R*^2^ coefficients of 0.94, 0.98, 0.98, 0.98, 0.94, 0.99, and 0.81, respectively, meaning that MZG3 has the best activity. Moreover, MZG3 exhibited the highest photocatalytic activity with illumination by visible light compared with the results of previous studies as illustrated in Table 2.

**Figure 7:**
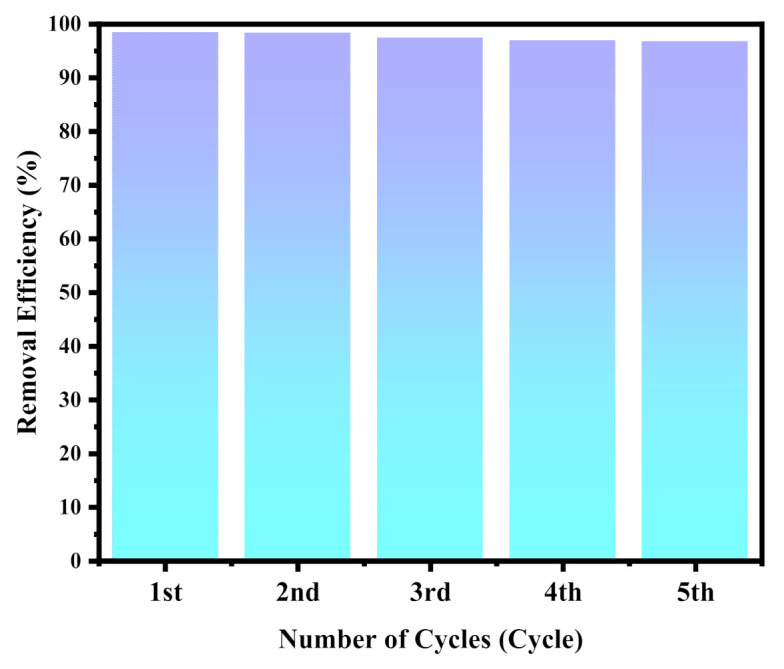
The MZG3 photocatalytic activity for degradation of organic pollutant remained stable (about 97%) throughout five consecutive cycles.

The stability and reusability of the MZG3 crystalline nanocomposite for organic pollutant degradation under visible light were tested several times as illustrated in Figure 7. The MZG3 crystalline nanocomposite was separated using an external magnet to be retested several times without causing any secondary pollutants. The functional groups in the MZG3 crystalline nanocomposite remained stable before and after its utilization as detected by FTIR and shown in Figure S3. Consequently, the MZG3 crystalline nanocomposite had perfect reusability with high recovery, without any secondary pollutants.

### 2.3. Photocatalysis Mechanism for Degradation of Organic Pollutant by MZG3 Crystalline Nanocomposites

**Figure 8:**
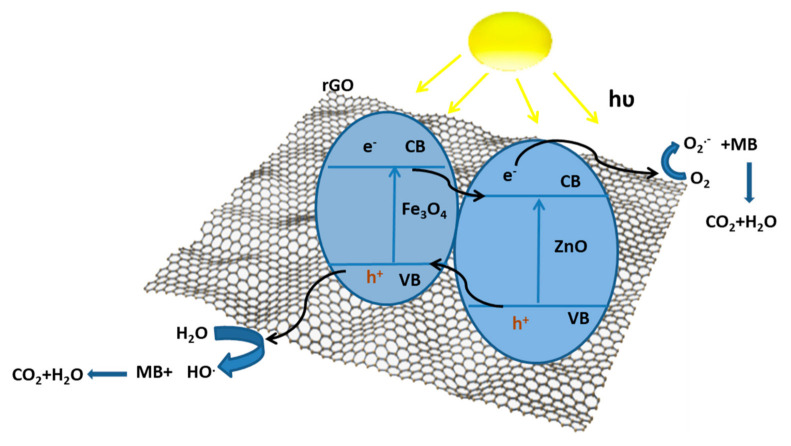
Schematic diagram of MB photodegradation by Fe_3_O_4_/rGO/ZnO crystalline nanocomposite under visible light.

The mechanism of photocatalysis degradation based on all the characterization results for the Fe_3_O_4_/rGO/ZnO crystalline nanocomposites presented in this part depended on all the previous results. The presence of Fe_3_O_4_ enhanced the light absorption of the catalyst in the visible region, and it had a small band gap energy. In addition, the junction between ZnO and Fe_3_O_4_ enhanced the stability between the electron/hole pairs. An important role of rGO was to capture the electrons from the conduction bands of ZnO and Fe_3_O_4_ to increase the lifetime of electrons; then, it enhanced the photocatalytic activity as recorded in this study. Thus, the mechanism of MZG3 was studied under visible light irradiation. It is summarized by Equations (4)–(11) and illustrated in the schematic graph (Figure 8).

The photocatalytic MZG3 can be excited under visible light irradiation to generate electron–hole pairs (Equation (5)). The electrons in the CB of Fe_3_O_4_ and ZnO were transferred to the surface of rGO. rGO acted as an electron acceptor and increased the formation of oxide radicals (O_2_**^.−^**) via a reduction process at the conduction band (Equation (6)). The oxide radicals played important roles for the degradation of organic pollutants and formation of hydroxyl radicals (Equation (7)). The holes in the valence band at ZnO with water molecules formed hydroxyl radicals (Equations (8)–(11)). Finally, the organic pollutant was degraded by hydroxyl radicals to environmentally friendly molecules: CO_2_ and H_2_O (Equation (12)).

Fe_3_O_4_/rGO/ZnO + hʋ → h^+^ + e^−^ (5)

Fe_3_O_4_/rGO(e^−^)/ZnO + O_2_ → O_2_·^−^ (6)

O_2_·^−^ + 2H_2_O → 4OH· (7)

h^+^+ H_2_O → OH·+ H^+^ (8)

H_2_O + H^+^ + O_2_·^−^ → H_2_O_2_ + OH· (9)

H_2_O_2_ + e^−^ → OH^−^ + OH· (10)

h^+^+ OH^−^ → OH· (11)

OH· + organic pollutant → degradation to CO_2_ + H_2_O (12)

## 3. Materials and Methods

### 3.1. Materials

Zinc nitrate hexahydrate (Zn(NO_3_)_2_·6H_2_O) (98%), ferrous sulfate heptahydrate (FeSO_4_·7H_2_O) (99.5%), iron chloride hexahydrate (FeCl_3_·6H_2_O) (97%), sodium hydroxide (NaOH) (98%), graphite fine powder (100%), potassium permanganate (KMnO_4_) (99%), hydrogen peroxide (H_2_O_2_) (30%), sulfuric acid (H_2_SO_4_) (95–98%), sodium nitrate (NaNO_3_) (99%), and methylene blue (MB) were acquired from Sigma-Aldrich (Munich, Germany). Ethanol was acquired from Honeywell organization with a high purity of 99.8%. All the purchased compounds were used as received with no further purification.

### 3.2. Nanoparticle Synthesis

#### 3.2.1. Synthesis of Crystalline ZnO Nanoparticles (Z)

ZnO nanoparticles (ZnO NPs) were synthesized using a precipitation method [61]. A 6 mM concentration of Zn(NO_3_)_2_·6H_2_O was made in 25 mL of distilled water with stirring for 30 min at 100 °C. The pH value was adjusted to 12 using 1 M NaOH and stirring for 1 h. The white precipitate was collected by centrifugation at a speed of 4000 rpm. The obtained precipitate was washed several times with deionized water and ethanol, and then dried at 60 °C for 6 h in an electric oven and calcined at 600 °C for 2 h in air.

#### 3.2.2. Synthesis of Crystalline Fe_3_O_4_ Magnetic Nanoparticles (M)

Fe_3_O_4_ magnetic nanoparticles (Fe_3_O_4_ NPs) were prepared using the coprecipitation method. We used ferrous and ferric salts at a 1:2 M ratio in the presence of N_2_ gas as recorded in our previous study [62]. The relevant chemical reaction can be expressed as follows:

Fe^2+^ + 2Fe^3+^ + 8OH^−^ → Fe_3_O_4_ + 4H_2_O (13)

#### 3.2.3. Synthesis of Crystalline Fe_3_O_4_/ZnO Nanocomposites (MZ)

MZ was prepared through a thermolytic process and calcination at 600 °C for 4 h. The optimum weight ratio of M:Z is 0.8:1 as deduced from the degradation of MB study presented in Figure S4.

#### 3.2.4. Synthesis of Graphene Oxide (GO)

GO was synthesized using a modified Hummer’s method as follows: A mixture of 1 g of graphite and 0.5 g of NaNO_3_ in 23 mL of H_2_SO_4_ was stirred for 15 min. Then, 5 g of potassium permanganate (KMnO_4_) was added slowly and gradually to oxidize the graphite into graphene oxide. Then, 50 mL of distilled water was added, and the mixture was kept under stirring for 2 h. Then, the temperature was increased to 98 °C for 30 min. Finally, the solution was treated with 60 mL of H_2_O_2_ to quench the oxidation reaction and to eliminate the intermediates and residual oxidants by reducing them into soluble components. The color became brown-yellow. It was washed with distilled water three times. The GO was dried at 60 °C for 12 h [41,62].

#### 3.2.5. Synthesis of Crystalline Fe_3_O_4_/rGO/ZnO Nanocomposites (MZG)

MZGx nanocomposite samples were prepared using a facile method. Graphene oxide was mixed with crystalline magnetic zinc oxide (MZ) by sonication for 30 min in aqueous solution. Different weight ratios of GO (0.5, 1, 1.5, and 2 wt %) MZG1, MZG2, MZG3, and MZG4, respectively, were synthesized to detect the best reduced graphene oxide loading [36,41].

### 3.3. Characterization

The phase of the prepared samples was examined through X-ray diffraction (XRD) using a diffractometer (Panalytical XPERT PRO MPD). CuKα radiation (λ = 1.5418 Å) was used at a rate of 40 kV and 40 mA. The structures of the prepared samples were investigated using Raman spectroscopy with a 532 nm laser source and 10 mW power (model Sentera, Bruker, Ettlingen, Germany). The functional groups were identified using a Fourier-transform infrared spectrometer (FTIR) model spectrum one (Perkin Elmer, Waltham, MA, USA) in the wave number range of 400 cm^−1^–4000 cm^−1^. The elemental composition of the nanocomposites was detected through X-ray photoelectron spectroscopy (XPS) using an Escalab 250 (Thermo Fisher Scientific Waltham, MA, USA) with a monochromatic Al K Alpha (1486.6 eV) at 2kV and 1 µA. Magnetic measurements were taken at room temperature using a vibrating sample magnetometer (VSM, 735VSM, Model 7410; Lake Shore, Westerville, OH, USA) with 31 kOe as a maximum magnetic field. The structure and morphology of the nanocomposites were detected by JEOL JEM 2100 (JEOL, Tokyo, Japan) high-resolution transmission electron microscopy (HRTEM) at an accelerating voltage of 200 kV. The optical reflectance was recorded using a UV–Vis spectrometer (Perkin Elmer Lambda 1050, Waltham, MA, USA). The photoluminescence spectra were recorded using a Cary Eclipse fluorescence spectrophotometer (Agilent Technologies, Santa Clara, CA, USA).

### 3.4. Photocatalytic Activity Study

The photocatalytic activity of the Fe_3_O_4_/rGO/ZnO nanocomposite was evaluated for the degradation of the molecules of methylene blue dye as a model of an organic pollutant under visible light irradiation. The experiments were carried out under visible light irradiation provided by a halogen lamp (500 W) at room temperature. The halogen lamp has an emission spectrum in the range 420–600 nm [37]. This light source was positioned at 10 cm from the cell. In each experiment, 50 mL of MB solution with an initial concentration of 100 ppm was taken in a glass beaker, and 0.1 g of the prepared catalyst was added. The mixed solution was magnetically stirred in the dark for 30 min until an adsorption/desorption equilibrium between the MB molecule and the catalyst was reached. Then, the experiment was continued with constant stirring under visible light irradiation for 90 min. The extent of the MB dye degradation was monitored using a UV–visible spectrophotometer (UV-1800, Shimadzu, Kyoto, Japan).

## 4. Conclusions

In this study, the Fe_3_O_4_/rGO/ZnO crystalline nanocomposite was optimized by different ratios of reduced graphene oxide. The photocatalytic performance of the Fe_3_O_4_/G/ZnO nanocomposite was investigated by the degradation of MB in aqueous solution under visible light irradiation. A high photocatalytic activity of about 98.5%, which stayed stable as the catalyst was used several times, was recorded for MZG3 due to the high absorption of the visible light in the presence of graphene layers and the enhancement by rGO of the stability between charge carriers. This high efficiency was reached at a certain amount of graphene (1.5 wt % for MZG3). However, this efficiency was slightly reduced over this optimum ratio of rGO due to the accumulation of graphene layers on the crystalline nanocomposite. MZG3 was easily separated without any secondary pollutants by an external magnetic field after the photodegradation process. This elaborated nanocomposite is promising for different applications in photocatalytic processes such as water splitting and dye-sensitized solar cells.

## Figures and Tables

**Table 1:** The band gap energy (Eg) values of all samples.

Sample Name	Eg_1_ (eV) for Fe_3_O_4_	Eg_2_ (eV) for ZnO
Z	-	3.20
MZ	2.07	3.19
MZG1	1.92	3.09
MZG2	1.91	3.02
MZG3	1.91	2.96
MZG4	1.89	2.97

**Table 2:** Photocatalytic degradation of MB under visible light with various photocatalysts.

Photocatalyst	Weight of Catalyst (g/L)	Concentration of MB (ppm)	Time(h)	Degradation (%)	Ref.
ZnS-TiO_2_/RGO	0.4	20	2	90	[56]
WO_3_/GO	0.5	3	1.2	82	[57]
Pt/WO_3_/GO	0.5	3	1.2	94	[57]
Fe_3_O_4_/CdWO_4_ +H_2_O_2_	0.1	20	2	32	[58]
Fe_3_O_4_/CdWO_4_/PrVO_4_ + H_2_O_2_	0.1	20	2	68	[58]
Fe_3_O_4_/ZnWO_4_/CeVO_4_ +H_2_O_2_	0.6	25	2	84	[59]
Pt/ZnO-MWCNT	0.4	100	1	74	[60]
MZG3	2	100	1.5	98.5	Our work

## Supplementary Materials

The following are available, Figure S1: Raman spectra of GO, Figure S2: VSM measurements for M and MZG3 nanocomposite, Figure S3: FTIR spectra of MZG3 nanocomposites before and after photocatalytic activity, Figure S4: The degradation of MB study under visible light for MZ1 (M:Z is 0.2:1), MZ2 (M:Z is 0.4:1), MZ3 (M:Z is 0.6:1), MZ4 (M:Z is 0.8:1), and MZ5 (M:Z is 1:1).

## Sample Availability

Samples of the compounds are available from the authors.
