# Supplementary material for: Magnetic ZnO Crystal Nanoparticle Growth on Reduced Graphene Oxide for Enhanced Photocatalytic Performance under Visible Light Irradiation
Source: Molecules. 2021 Apr 14;26(8):2269. doi: 10.3390/molecules26082269 (PMC8070817; doi:10.3390/molecules26082269)
Supplement: Supplementary file 1 [file molecules-26-02269-s001.pdf]

Magnetic ZnO crystals nanoparticles growth on reduced graphene oxide for enhanced photocatalytic performance under visible light irradiation

Rania Elshypany [1], Hanaa Selim [1], K. Zakaria [1], Ahmed H. Moustafa [2], Sadeek. A. Sadeek [2], S.I. Shara [1], Patrice Raynaud [3], Amr A. Nada [1,*]

[1] Department of Analysis and Evaluation, Egyptian Petroleum Research Institute, Nasr City, Cairo, 11727, Egypt.
[2] Department of Chemistry, Faculty of Science, Zagazig University, Zagazig, 44519 Egypt.
[3] Laboratoire Plasma et Conversion d'Energie (LAPLACE), Université de Toulouse, CNRS, INPT, UPS, 31062, Toulouse, France.
* Correspondence: amr.nada@epri.sci.eg, amr.nada@laplace.univ-tlse.fr and chem_amr@yahoo.com.

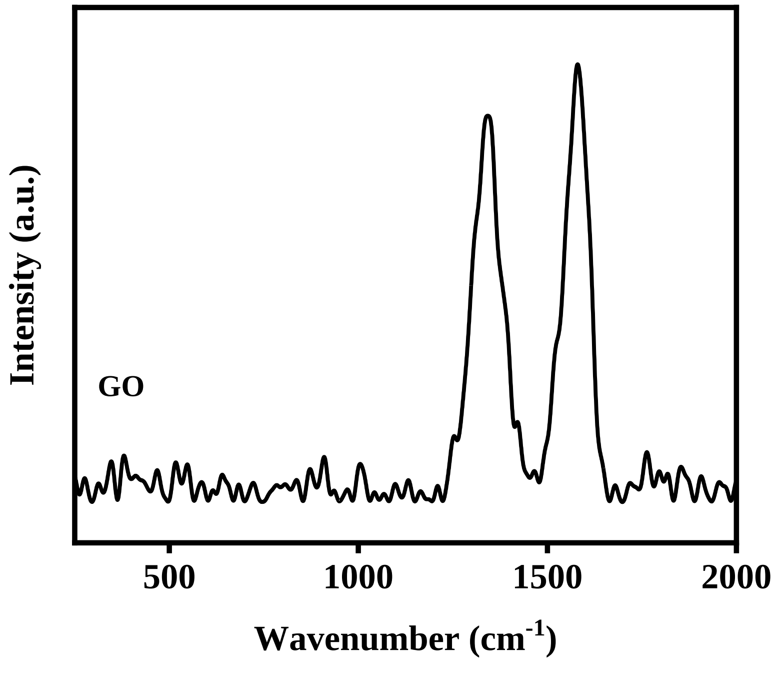

Figure S1. Raman spectra of GO.

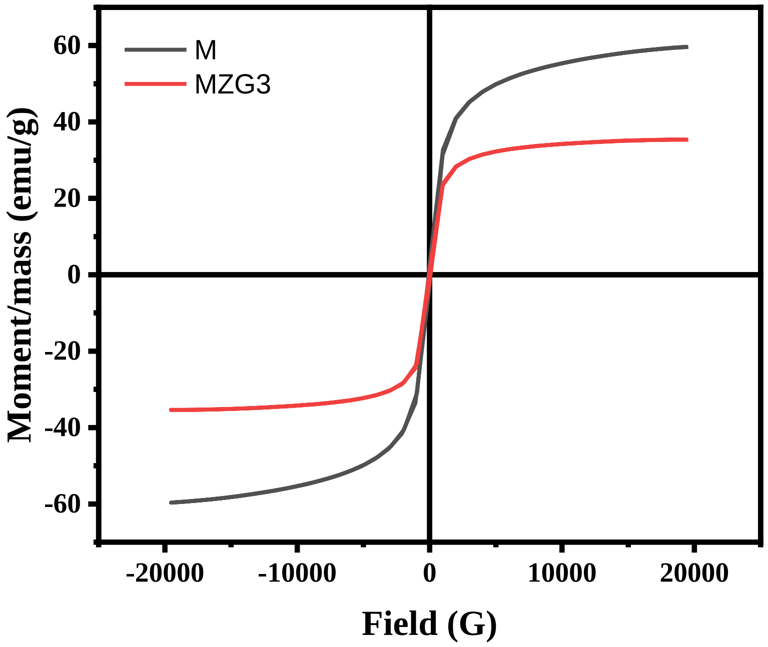

*

Figure S2. VSM measurements for M and MZG3 nanocomposite.

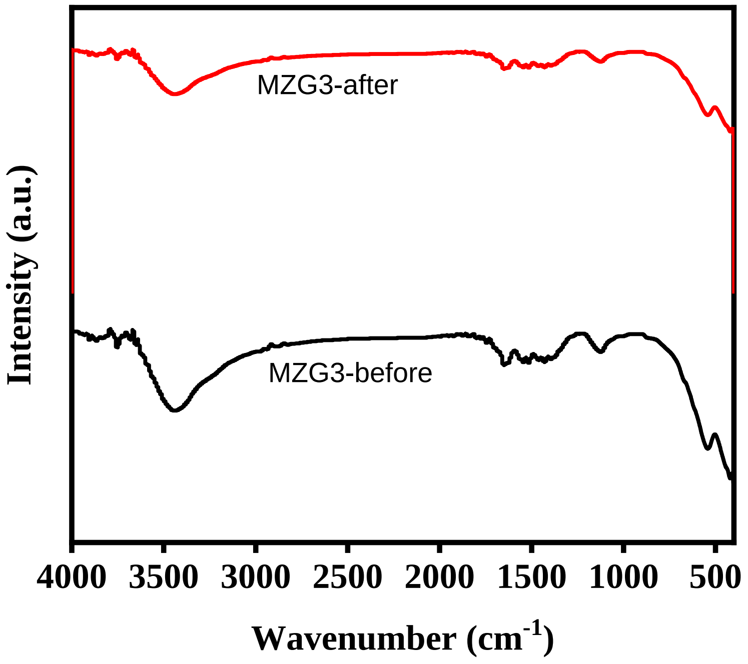

Figure S3. FT-IR spectra of MZG3 nanocomposites before and after of photocatalytic activity.

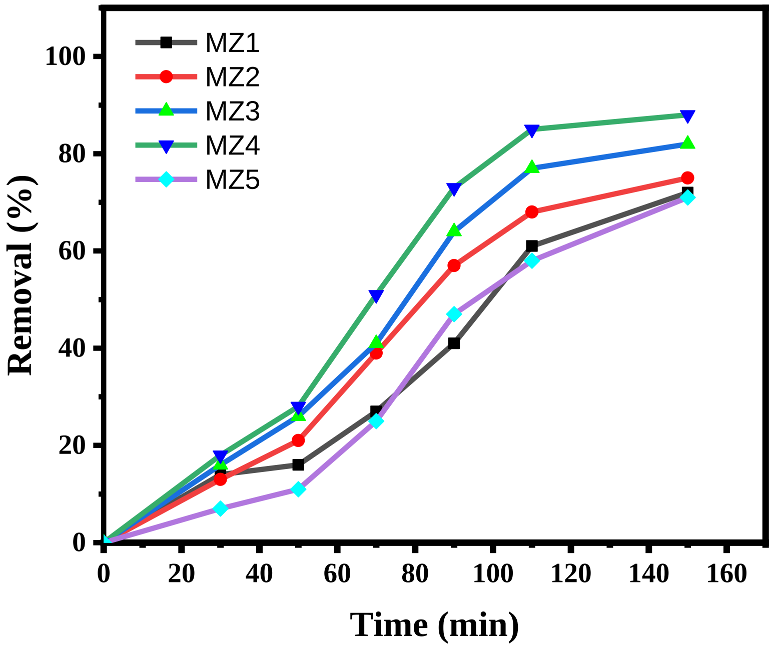

Figure S4. The degradation of MB study under visible light for MZ1 (M:Z is 0.2:1), MZ2 (M:Z is 0.4:1), MZ3 (M:Z is 0.6:1), MZ4 (M:Z is 0.8:1) and MZ5 (M:Z is 1:1).
